# Predictive modeling of Time-Temperature-Transformation diagram of metallic glasses based on atomistically-informed classical nucleation theory

**DOI:** 10.1038/s41598-017-06482-8

**Published:** 2017-08-03

**Authors:** Yuji Sato, Chiaki Nakai, Masato Wakeda, Shigenobu Ogata

**Affiliations:** 1 Department of Mechanical Science and Bioengineering, Graduate School of Engineering Science, Osaka University, Osaka, 560-8531 Japan; 2 Center for Elements Strategy Initiative for Structural Materials (ESISM), Kyoto University, Kyoto, 606-8501 Japan

## Abstract

Theoretical prediction of glass forming ability (GFA) of metallic alloys is a key process in exploring metallic alloy compositions with excellent GFA and thus with the ability to form a large-sized bulk metallic glass. Molecular dynamics (MD) simulation is a promising tool to achieve a theoretical prediction. However, direct MD prediction continues to be challenging due to the time-scale limitation of MD. With respect to practical bulk metallic glass alloys, the time necessary for quenching at a typical cooling rate is five or more orders of magnitude higher than that at the MD time-scale. To overcome the time-scale issue, this study proposes a combined method of classical nucleation theory and MD simulations. The method actually allows to depict the time-temperature-transformation (TTT) diagram of the bulk metallic glass alloys. The TTT directly provides a prediction of the critical cooling rate and GFA. Although the method assumes conventional classical nucleation theory, all the material parameters appearing in the theory were determined by MD simulations using realistic interatomic potentials. The method is used to compute the TTT diagrams and critical cooling rates of two Cu-Zr alloy compositions (Cu_50_Zr_50_ and Cu_20_Zr_80_). The results indicate that the proposed method reasonably predicts the critical cooling rate based on the computed TTT.

## Introduction

Metallic glasses possess brilliant properties as structural materials including high elastic limit^1^, high toughness^2^, and high corrosion resistance^3^. However, since reachable size of bulk metallic glass has been limited within centimeters even for highly selected alloy compositions, the application of metallic glasses is substantially restricted as structural materials. Alloys with a lower glass forming ability (GFA) require a higher cooling rate in the melt-quenching process to realize a glass state eventually. In other words, the quenching must be finished prior to the spontaneous nucleation of a critical-sized crystal nucleus in the molten alloy. The critical size is the minimum size required for thermodynamic downhill crystal growth. A critical cooling rate ${\dot{T}}_{{\rm{c}}}$ is defined as the minimum cooling rate that results in a glass state. When we quench a plate-like shaped molten alloy with thickness *L* from very high temperature *T*_i_ to ambient temperature *T*_s_, the spatial distribution of time dependent cooling rate in the molten alloy $\dot{T}(x,t)$ could be derived by solving the 1D heat conduction equation:

1 $$\frac{\partial T}{\partial t}=\alpha \frac{{\partial }^{2}T}{\partial {x}^{2}},$$

under the conditions *T*(*x*, 0) = *T*_i_ and *T*(0, *t*) = *T*(*L*, *t*) = *T*_s_, where *t* denotes time, *x* (0 ≤ *x* ≤ *L*) denotes coordinate of out-of-plane direction, and *α* denotes thermal diffusivity. Assuming constant thermal diffusivity, the solution is (see Supplemental Information for the details)

2 $$\dot{T}(x,t)=\frac{4\pi \alpha ({T}_{{\rm{i}}}-{T}_{{\rm{s}}})}{{L}^{2}}\sum _{m=0}^{{\rm{\infty }}}(2m+1)\exp [-\alpha {\{\frac{(2m+1)\pi }{L}\}}^{2}t]\sin \{\frac{(2m+1)\pi }{L}x\}.$$

Since $\dot{T} > {\dot{T}}_{{\rm{c}}}$ should be always satisfied throughout the molten alloy sample to obtain a glass state, the critical cooling rate ${\dot{T}}_{{\rm{c}}}$ actually limits the possible sample size *L* as it can be understood from Eq. (2). Thus, ${\dot{T}}_{{\rm{c}}}$ is potentially a good measure of GFA^4^. The key to obtaining a larger sample size involves searching for alloy composites with a slower critical cooling rate. The critical cooling rate can be estimated after depicting the time-temperature-transformation (TTT) diagram of an alloy^5^. Therefore, it is necessary to establish a method to predicting the TTT diagram as it can lead to a computational high-throughput screening of alloy composition to obtain a larger bulk metallic glass sample. Furthermore, it is essential to compute the incubation time for the nucleation of the critical crystal nucleus to illustrate the TTT^5^. Currently, molecular dynamics (MD) simulation is the best tool available for the computation of the incubation time because the critical crystal nucleus typically corresponds to the nanometer range^6^, and thus it is necessary for the crystal nucleation process to consist of atomic scale events. Additionally, the quantitative analyses of the event properties are possible given that reliable interatomic potentials are provided. Recently, a study demonstrated solidification from melting by using direct MD quenching simulations^7, 8^. This is followed by estimating the TTT and critical cooling rate from direct MD observation of the incubation time. However, these studies only focused on highly simplified model materials, such as the Lennard-Jones system, which possess a very high critical cooling rate because of the time scale limitations of MD simulations. Conversely, the composition of target alloys must possess the potential to generate a centimeter sized metallic glass with a very long incubation time and a very slow critical cooling rate when compared to simple system models. Thus, it is necessary for the incubation time to considerably exceed typical MD time scales such as microseconds. Thus, a direct MD simulation does not work well due to the fore-mentioned limitation. Therefore, in this study, classical nucleation theory is employed to compute the incubation time as opposed to the direct MD simulations. However, the intrinsic parameters of the materials that appear in classical nucleation theory are determined using MD simulations such as the free energy difference between a melt and crystal and the interfacial free energy between a melt and crystal. Finally, the TTT diagram is depicted, and the critical cooling rate is evaluated based on the TTT diagram.

## Results and Discussion

Two alloy compositions possessing different GFA, such as Cu_50_Zr_50_ and Cu_20_Zr_80_, are examined in the study to demonstrate the proposed method.

**Figure 1:**
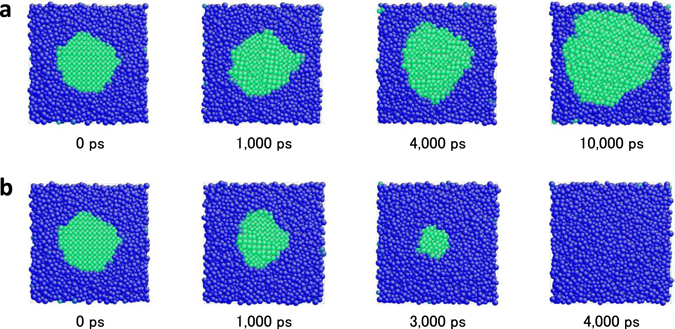
Snapshots of crystal nucleus growth and shrink processes of Cu_50_Zr_50_ model. (**a**) denotes the growth process (*T* = 1,100 K, *r* = 2.0 nm), and (**b**) denotes the shrink process (*T* = 1,200 K, *r* = 2.0 nm). Green atoms represent the crystal phase while blue atoms represent the liquid phase detected using bond-order analysis^11^.

**Figure 2:**
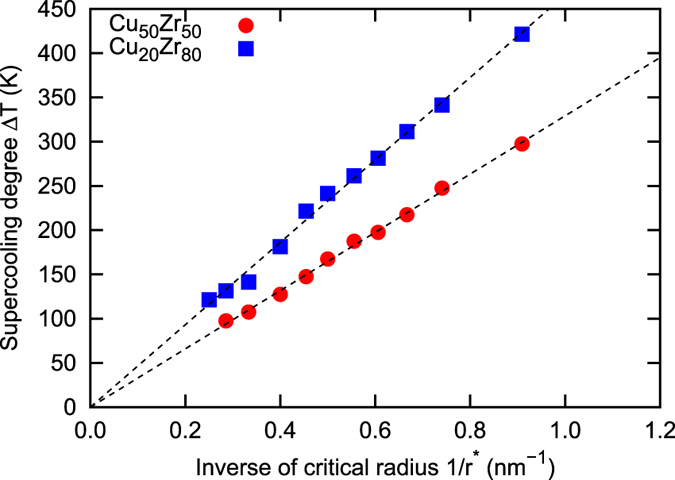
The relationship between supercooling degree Δ*T* and inverse of the critical radius 1/r*.

Firstly, the critical nucleation radius r* is determined as follows. A cubic simulation cell is set with a periodic boundary condition (PBC) containing *N* atoms, which consist of supercooled liquid (melt) and a spherical crystal nucleus of a radius *r* (see Fig. S1 and Tables S1 and S2 for the details). The Finnis-Sinclair (FS) potential^9^ is used to describe the interatomic interaction for both alloy systems. The B2 crystal structure is assumed as the crystal structure of Cu_50_Zr_50_^10^, while a distorted BCC like structure determined by MD quenching simulation is used as the crystal structure of Cu_20_Zr_80_. The melts of both alloys are prepared by annealing at 2,500 K for 100 ps under a zero pressure condition. The crystal nucleus is then embedded into the melt, while omitting all atoms in the melt that overlap with the embedded crystal nucleus. The systems are subsequently quenched at 10 K for 2 ps to fill the space gap at the interface between the melt and the crystal nucleus. The MD time step of 2 fs is used throughout the study for all the MD simulations. The constructed melt-crystal models are employed as the initial configuration to determine the temperature dependent critical nucleus radius r*(*T*). The NPT ensemble MD simulations are performed with respect to the models with different nucleus radius at different temperatures under a zero pressure condition to determine the critical temperature as the middle point between the highest nucleus growth and the lowest nucleus shrinking temperatures (see Fig. S2). Ten MD simulations are performed for each condition to reduce the statistical error. Actual growing and shrinking behaviors of the Cu_50_Zr_50_ crystal nucleus of *r* = 2.0 nm immediately above and below the critical temperature *T* = 1,175 K are shown in Fig. 1. The atoms in the melt and crystal are identified and colored using bond-order analysis^11^. The relationship between temperature and inverse of radius is summarized in Fig. S2, which clearly shows a trend in which the inverse of critical radius approximately linearly increases with increases in the temperature. Figure 2 represents the relationship between supercooled degree Δ*T* = *T*_m_ − *T* and the inverse of r*. The results indicate that Δ*T* is reasonably assumed as proportional to the inverse of r*:

3 $${\rm{\Delta }}T={T}_{{\rm{m}}}-T\simeq \frac{k}{{r}^{\ast }},$$

where the values of *k* are estimated as −3.29 × 10^2^ nm/K (Cu_50_Zr_50_) and −4.66 × 10^2^ nm/K (Cu_20_Zr_80_), respectively. The melting temperatures *T*_m_ are determined from the ordinate intercept of the linear fitting line for critical temperatures in Fig. S2. Since Eq. (3), *T*_m_ is assumed as the critical temperature at r* → ∞. The obtained melting temperature is shown in Table 1. The melting temperature of Cu_50_Zr_50_ is lower than that of Cu_20_Zr_80_ by 100 K. The difference is consistent with an experiment involving Cu-Zr alloys^12^ while the actual melting temperature of Cu_50_Zr_50_, *T*_m_ = 1,208 K^13^ is slightly lower (by 134 K) than the computed value. The discrepancy can be mainly attributed to an underestimation of melting temperature subject to FS potential energetics. With respect to Cu_50_Zr_50_, the melting temperature is also estimated using a melt-crystal biphase model with PBC and a flat interface that is perpendicular to the (100) crystal plane (see Fig. S3). Determination of temperature involves an immobile interface under NPH ensemble MD simulation at a zero pressure condition^14^ to provide the melting temperature. Although the melting temperature determined using biphase model can depend on interfacial crystal orientation, both are in reasonable agreement with each other as shown in Fig. S2(a).

**Table 1:** Obtained values of the melting temperature and latent heat.

alloy	*T*_m_ (K)	Δ*H*_m_ (J m^−3^)
Cu_50_Zr_50_	1,342	1.9 × 10^9^
Cu_20_Zr_80_	1,436	7.4 × 10^8^

The free energy barrier of crystal nucleation ΔG* is computed using Eq. (16). The interfacial free energy *σ* is computed using Eqs. (14) and (15). The latent heat per unit volume Δ*H*_m_ in Eq. (15) is evaluated from the enthalpy difference between the melt and crystal parts in the melt-crystal biphase model (Fig. S3). The melt and crystal models used in the biphase model are obtained by annealing the melt for 100 ps at 2,500 K and relaxing the crystal structure for 100 ps at 0.1 K under a zero pressure condition, respectively. The two structures are subsequently attached as shown in Fig. S3, and 600 ps MD relaxation is performed at the melting temperature *T*_m_ and zero pressure condition. The computed Δ*H*_m_ values are shown in Table 1. The findings reveal that Δ*H*_m_ of Cu_50_Zr_50_ is almost twice that of Cu_20_Zr_80_. The temperature dependent enthalpy is obtained by averaging the total energy over a 1 ns NPT ensemble MD simulation using a *N* = 16,000 atoms simulation cell with PBC at different temperatures ranging from 50 K to *T*_m_ with 50 K intervals and a zero pressure condition. The obtained temperature dependent enthalpies of the melt and crystal are fitted by the following quadratic polynomial function (see Fig. S4):

4 $$H(T)\simeq a{T}^{2}+bT+c.$$

The volumetric specific heats *C*_p_ of the melt and crystal are then obtained as the derivative of enthalpy (Eq. (4)) with respect to the temperature at constant pressure as follows:

5 $${C}_{{\rm{p}}}={(\frac{\partial H}{\partial T})}_{{\rm{p}}}\simeq 2aT+b\mathrm{.}$$

**Figure 3:**
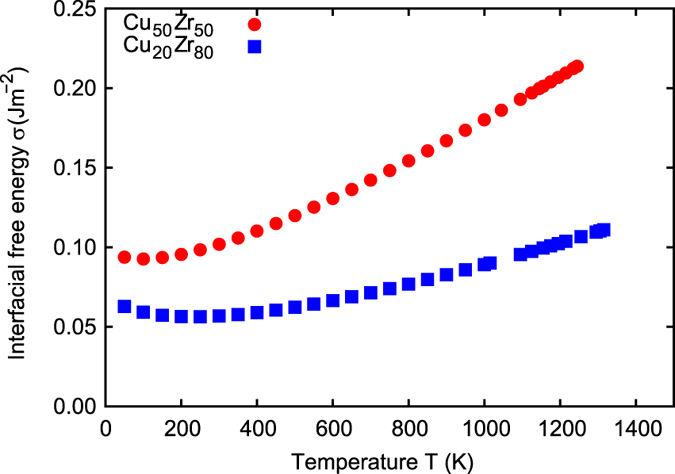
Temperature dependent interfacial free energy.

Additionally, Δ*C*_p_ in Eq. (14) is estimated by ${\rm{\Delta }}{C}_{{\rm{p}}}={C}_{{\rm{p}}}^{{\rm{melt}}}-{C}_{{\rm{p}}}^{{\rm{crystal}}}$. The parameters determined in Eqs. (4) and (5) are summarized in Table S3. Subsequently, the temperature dependent interfacial free energy *σ* is computed and shown in Fig. 3. The interfacial free energy of Cu_50_Zr_50_ is consistently higher than that of Cu_20_Zr_80_.

**Figure 4:**
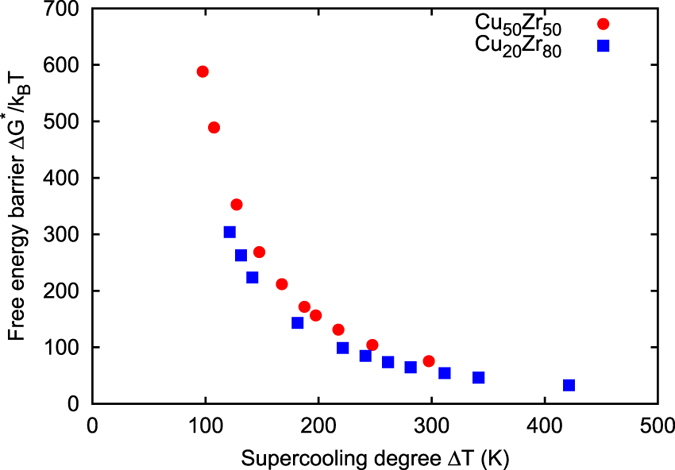
Nucleation free energy barrier as a function of Δ*T*.

Eventually, the free energy barrier of crystal nucleation ΔG* is computed as a function of temperature and shown in Fig. 4.

**Figure 5:**
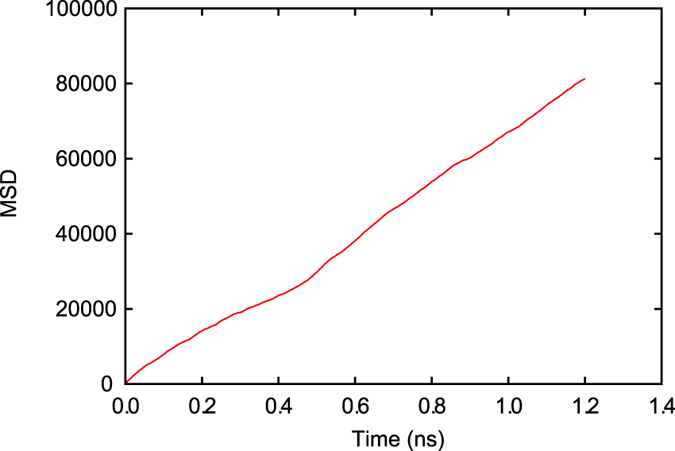
Time evolution of MSD 〈Δn*^2^(*t*)〉 at *T* = 1,155 K (Cu_50_Zr_50_).

**Figure 6:**
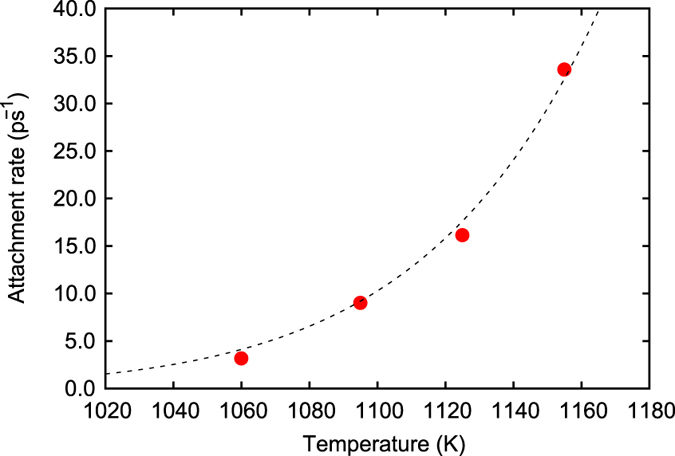
Attachment rate - temperature relationship (Cu_50_Zr_50_). The dashed line represents the fitted curve (Eq. (6) and Table S4).

The temperature dependent nucleation rate *J* and the incubation time *t* are directly computed from Eq. (8). The atomic number density of the melt *ρ*_melt_ and that of the critical crystal nucleus ${\rho }_{{\rm{crystal}}}^{\ast }$ are computed and summarized in Table 2 by taking the average volume of simulation cell *V* (*N* = 16,000) with PBC over 100 ps NPT ensemble MD simulation at the critical temperature and a zero pressure condition. The Zeldovich factor *Z* is estimated by using Eqs. (18) and (19). With the exception of the parameters obtained in the previous sections, the attachment rate *f* ^+^ that appears in the pre-exponential factor *J*_0_ (Eq. (19)) is still unknown, and thus it is necessary to obtain *J*. In order to compute the attachment rate, 4 ns NPT ensemble MD simulations on the same melt - spherical crystal model with *r* = r*(*T*) used in the previous analysis are performed at different temperatures ranging from 1,060 to 1,160 K under a zero pressure condition. The number of atoms belonging to the embedded crystal, Δn*(*t*) = n*(*t*) − n*(0), are recorded during the MD simulations. This is followed by obtaining the MSD 〈Δn*^2^(*t*)〉 and attachment rate (Eq. (20)). It should be noted that ten independent MD simulations are performed for each temperature, and they are averaged to reduce the statistical error. Figure 5 shows time evolution of the MSD for Cu_50_Zr_50_ at *T* = 1,155 K. The slope of the MSD-time plot corresponds to twice that of the attachment rate, 2*f* ^+^(n*). The computed attachment rates are plotted in Fig. 6 for Cu_50_Zr_50_ and are summarized for both alloys in Table 2. The attachment rate increases with increases in the temperature. The attachment rate of Cu_20_Zr_80_ is an order of magnitude higher than that of Cu_50_Zr_50_. The computed attachment rate is used to eventually compute the nucleation rate *J* of unit volume and summarize it in the Table 2. The nucleation rate rapidly increases with increases in the temperature. The rapid change in the nucleation rate is mainly attributed to the exponential term in Eq. (19) as opposed to the pre-exponential factors. As previously stated, the direct MD analyses of the attachment rate *f* ^+^(n*) are only performed above *T* = 1,060 K because a large statistical error due to the lower number of samples is expected below this temperature. In order to predict the attachment rate at low temperatures, the following Arrhenius type exponential function fitted to high temperature MD data of the attachment rate is used instead of performing direct MD analysis at the low temperatures.

6 $${f}^{+}({n}^{\ast })\simeq A\exp (-B/T),$$

where *A* and *B* denote fitting parameters. The values of *A* and *B* are summarized in Table S4. It is worth noting that recent theoretical crystallization study for a Lennard-Jones system^15^ shows a super-Arrhenius behavior of the attachment rate. Actually our attachment rate data of Cu_20_Zr_80_ also seem to exhibit super-Arrhenius like behavior, while data of Cu_50_Zr_50_ do not clearly exhibit at least within the examined temperature range (see Fig. S5). However, since the deviation from Arrhenius behavior is not significant, here the Arrhenius behavior was assumed for simplicity.

**Table 2:** Obtained parameters with respect to the nucleation rate.

alloy	*T* (K)	${{\boldsymbol{\rho }}}_{{\bf{c}}}^{{\boldsymbol{\ast }}}$ (m^−3^)	*ρ*_m_ (m^−3^)	*Z*	*f* ^+^(n*) (ps^−1^)	log_10_ exp(−ΔG*/*k*_B_ *T*)	log_10_ *J*/Ω (m^−3^s^−1^)
Cu_50_Zr_50_	1,060	5.73 × 10^28^	5.41 × 10^28^	4.5 × 10^−3^	3.1	−36	3
′′	1,095	5.72 × 10^28^	5.40 × 10^28^	3.2 × 10^−3^	9.0	−43	−4
′′	1,125	5.72 × 10^28^	5.39 × 10^28^	2.6 × 10^−3^	16.2	−52	−13
′′	1,155	5.71 × 10^28^	5.38 × 10^28^	1.8 × 10^−3^	33.6	−77	−37
Cu_20_Zr_80_	1,095	4.68 × 10^28^	4.58 × 10^28^	2.6 × 10^−3^	1.40 × 10^2^	−21	19
′′	1,125	4.67 × 10^28^	4.57 × 10^28^	2.2 × 10^−3^	1.63 × 10^2^	−26	14
′′	1,155	4.67 × 10^28^	4.57 × 10^28^	1.8 × 10^−3^	1.77 × 10^2^	−31	9
′′	1,175	4.66 × 10^28^	4.57 × 10^28^	1.5 × 10^−3^	1.78 × 10^2^	−37	3
′′	1,195	4.66 × 10^28^	4.56 × 10^28^	1.2 × 10^−3^	1.86 × 10^2^	−47	−7

**Figure 7:**
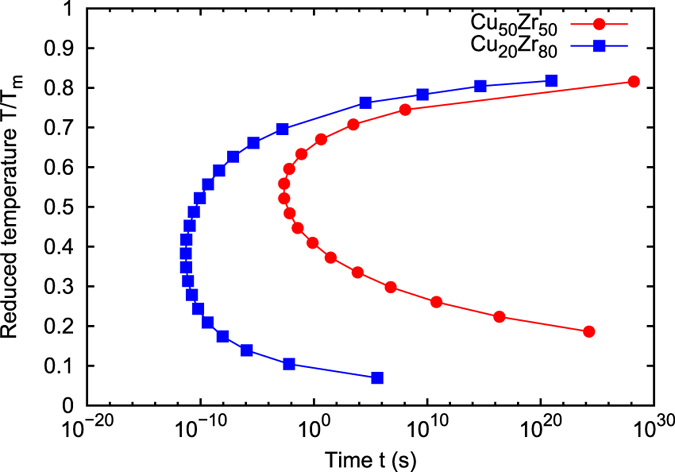
TTT diagrams of Cu_50_Zr_50_ and Cu_20_Zr_80_.

The TTT diagrams for Cu_50_Zr_50_ and Cu_20_Zr_80_ are depicted with respect to the temperature dependent incubation time *t* = 1/*J* using the parameters obtained in previous analysis and shown in Fig. 7. The critical cooling rate ${\dot{T}}_{{\rm{c}}}$ is approximately calculated from the following equation:

7 $${\dot{T}}_{{\rm{c}}}\simeq \frac{{T}_{{\rm{m}}}-{T}_{{\rm{n}}}}{{t}_{{\rm{n}}}},$$

where *T*_n_ and *t*_n_ denote the nose temperature of the TTT diagram and incubation time at the nose temperature, respectively. Table 3 shows the estimated critical cooling rate for a local volume of Ω = 343 nm^3^. Fortunately, with respect to Cu_20_Zr_80_, the crystallization can be observed even in a direct MD quenching simulation due to its very low incubation time. Therefore, the obtained critical cooling rate using the proposed method is compared with that using direct MD simulation for the same average volume of the simulation cell. In order to directly estimate the critical cooling rate from MD, 1 ns NPT ensemble MD simulations are performed using *N* = 16,000 atoms simulation cell with PBC at different temperatures ranging from 800 K to *T*_m_ with 50 K intervals. The incubation time for each temperature is estimated as an incubation time for a rapid reduction of system potential energy, which is a sign of crystallization. The nose temperature corresponds to *T*_n_ = 950 K, and the incubation time corresponds to *t*_n_ = 4.5 × 10^−10^ s. The average volume of the simulation cell at *T*_n_ corresponds to Ω = 343 nm^3^. The critical cooling rates obtained in the direct MD and experiment are also shown in Table 3. The experimental critical cooling rate ${\dot{T}}_{{\rm{c}}}$ is approximately estimated using Eq. (2) as the slowest cooling rate throughout the sample when the local temperature $T(x,{t}_{{\rm{n}}}(x))={T}_{{\rm{n}}}\simeq ({T}_{{\rm{m}}}+{T}_{{\rm{g}}})/2;\,\,{\dot{T}}_{{\rm{c}}}={min}_{x\in L}(\dot{T}(x,{t}_{{\rm{n}}}(x)))$, *t*_n_(*x*): time when *T*(*x*, *t*_n_(*x*)) = *T*_n_ at *x*, with *L* = 2 mm^16^, *α* = 2.5 × 10^−2^ cm^2^/s^17^, *T*_m_ = 1,208 K^13^, *T*_m_ − *T*_g_ = 400 K^17^, *T*_i_ = 1,500 K (>*T*_m_), *T*_s_ = 300 K (room temperature) and assuming that the volume with the slowest cooling rate in the system is the same as the volume of the simulation cell (Ω = 343 nm^3^) (see Fig. S6). Note that the order of the slowest cooling rate has a weak dependence on the temperature *T*. The results in Table 3 indicate that the proposed method can reasonably reproduce the critical cooling rate obtained by the direct MD simulation and experiment. The computed critical cooling rate of Cu_50_Zr_50_ is significantly lower than that of Cu_20_Zr_80_. This corresponds to the fact that Cu_50_Zr_50_ does actually form a bulk metallic glass at the usual experimental cooling rate^16^. In contrast, Cu_20_Zr_80_ does not form a bulk metallic glass even at highest possible cooling rate in the experiment. Hence, the proposed classical nucleation theory - MD combined method allows the prediction of the critical cooling rate.

**Table 3:** Critical cooling rate for a local volume corresponding to Ω = 343 nm^3^.

alloy	log_10_(${\dot{{\boldsymbol{T}}}}_{{\bf{c}}}$ (K/s))
Cu_50_Zr_50_ (This study)	5
Cu_50_Zr_50_(Experiment^16^)	∼4
Cu_20_Zr_80_ (This study)	14
Cu_20_Zr_80_ (Direct MD)	12

It should be noted that a homogeneous nucleation is assumed in this study. However, in practice, the nucleation is mostly heterogeneous due to the existence of impurities and/or the wall surfaces of containers. Thus, the proposed method underestimates the critical cooling rate. Nevertheless, it is necessary to adequately reproduce the GFA ranking, which is the most important factor in the high-throughput alloy design. It is worth noting that the target crystal structure and interatomic potential function are usually unknown for less familiar alloys. Although determination of them is non-straightforward task, recent development of crystal structure prediction methods^18, 19^ and interatomic potential construction methods^20, 21^ from first-principles may permit the task.

In this study, an atomistically-informed method is proposed to predict a TTT diagram of alloys over a wide temperature range by combining classical nucleation theory and MD simulations. The proposed method is used to depict the TTT diagram and critical cooling rate of two Cu-Zr alloys, namely Cu_50_Zr_50_ and Cu_20_Zr_80_. The results indicate that the method reasonably reproduces the GFA ranking and the critical cooling rate by comparing the direct MD simulation and experimental knowledge. Hence, it is expected that the method can open up a computational high-throughput screening of higher GFA alloys.

## Methods

The incubation time *t* corresponds to the inverse of nucleation rate *J* for a certain piece of volume Ω^22^ of melt, which is subject to the Arrhenius equation^23^ as follows:

8 $$J={\rm{\Omega }}{J}_{0}\exp (-\frac{{\rm{\Delta }}{G}^{\ast }}{{k}_{{\rm{B}}}T}),$$

where *k*_B_ denotes the Boltzmann constant, and *T* denotes temperature of the supercooled melt. Additionally, ΔG* denotes the free energy barrier of the nucleation process of a critical sized crystal nucleus. Subject to the classical nucleation theory, ΔG* is formulated as follows.

A change in the Gibbs free energy during the crystal growth process Δ*G* is expressed as a function of crystal nucleus radius *r* and interfacial free energy *σ* subject to the approximation of spherical nucleus shape^24^ as follows:

9 $${\rm{\Delta }}G=-\frac{4\pi {r}^{3}}{3}{\rm{\Delta }}{G}_{{\rm{v}}}+4\pi {r}^{2}\sigma ,$$

where Δ*G*_v_ denotes free energy difference per unit volume between the melt and crystal. Extant studies proposed several approximate expressions of Δ*G*_v_^25, 26^. In this study, it is directly computes by using atomistically computed isobaric heat capacity per unit volume (volumetric specific heat) of melt ${C}_{{\rm{p}}}^{{\rm{melt}}}$ and crystal ${C}_{{\rm{p}}}^{{\rm{crystal}}}$. The Δ*G*_v_ is given as follows:

10 $${\rm{\Delta }}{G}_{{\rm{v}}}={\rm{\Delta }}H-T{\rm{\Delta }}S,$$

where Δ*H* and Δ*S* denote enthalpy and entropy differences per unit volume between the melt and crystal, respectively. The Δ*H* and Δ*S* can be formulated using the isobaric volumetric specific heat difference between the melt and crystal, ${\rm{\Delta }}{C}_{{\rm{p}}}={C}_{{\rm{p}}}^{{\rm{melt}}}-{C}_{{\rm{p}}}^{{\rm{crystal}}}$ as follows:

11 $${\rm{\Delta }}H={\rm{\Delta }}{H}_{{\rm{m}}}-{\int }_{T}^{{T}_{{\rm{m}}}}{\rm{\Delta }}{C}_{{\rm{p}}}{\rm{d}}T,$$

12 $${\rm{\Delta }}S={\rm{\Delta }}{S}_{{\rm{m}}}-{\int }_{T}^{{T}_{{\rm{m}}}}\frac{{\rm{\Delta }}{C}_{{\rm{p}}}}{T}{\rm{d}}T,$$

where Δ*H*_m_ denotes melting enthalpy (latent heat), and Δ*S*_m_ denotes melting entropy. These are related to each other as follows:

13 $${\rm{\Delta }}{S}_{{\rm{m}}}=\frac{{\rm{\Delta }}{H}_{{\rm{m}}}}{{T}_{{\rm{m}}}}\mathrm{.}$$

Substituting Eqs. (11) and (13) into Eq. (10), the following expression is obtained:

14 $${\rm{\Delta }}{G}_{{\rm{v}}}={\rm{\Delta }}{H}_{{\rm{m}}}(1-\frac{T}{{T}_{{\rm{m}}}})-{\int }_{T}^{{T}_{{\rm{m}}}}{\rm{\Delta }}{C}_{{\rm{p}}}{\rm{d}}T+T{\int }_{T}^{{T}_{{\rm{m}}}}\frac{{\rm{\Delta }}{C}_{{\rm{p}}}}{T}{\rm{d}}T\mathrm{.}$$

It should be noted that the crystal nucleus shape is not exactly spherical as described by like Wulff construction^27^ because the interfacial energy is typically anisotropic and additionally, the interface should not very smooth at the atomic level, and thereby it should not be possible to precisely describe the interface by a smooth function especially with respect to a very small crystal nucleus. Specifically, this fact is observed in atomic simulations^28^ and also observed in MD simulations (see Fig. S1). Nevertheless, the spherical approximation is sufficient for the incubation time estimation as demonstrated later in the study.

In the right hand side of Eq. (9), the two terms compete with each other, and this leads to a crossover with respect to the crystal nucleus radius *r*. The crystal nucleus tends to shrink at a small *r* (dΔ*G*/d*r* > 0) while the crystal nucleus tends to grow spontaneously at a large *r* (dΔ*G*/d*r* < 0). The first term decreases in proportion to *r* ^3^ while the second increases in proportion to *r* ^2^ with increases in *r*. Therefore, Δ*G* is maximized at a critical nucleus radius *r* = r*, which should satisfy the following:

15 $${(\frac{{\rm{d}}{\rm{\Delta }}G}{{\rm{d}}r})}_{r={r}^{\ast }}=-4\pi {r}^{\ast 2}{\rm{\Delta }}{G}_{{\rm{v}}}+8\pi {r}^{\ast }\sigma =0.$$

The free energy barrier, which corresponds to energy at the critical nucleus radius, is given as follows:

16 $${\rm{\Delta }}{G}^{\ast }={\rm{\Delta }}{G}_{r={r}^{\ast }}=\frac{4}{3}\pi \sigma {r}^{\ast 2}.$$

It is assumed that the nucleus growth and shrink are achieved by attaching atoms to the nucleus, and thus the pre-exponential factor *J*_0_ in Eq. (8) is expressed as follows^29^:

17 $${J}_{0}={\rho }_{{\rm{m}}}Z{f}^{+}({n}^{\ast }),$$

18 $$Z=\sqrt{-\frac{1}{2\pi {k}_{{\rm{B}}}T}{(\frac{{\partial }^{2}{\rm{\Delta }}G}{\partial {n}^{2}})}_{n={n}^{\ast }}},$$

19 $${(\frac{{{\rm{\partial }}}^{2}{\rm{\Delta }}G}{{\rm{\partial }}{n}^{2}})}_{n={n}^{\ast }}=-\frac{8}{27}\pi \sigma {(\frac{3}{4\pi {\rho }_{{\rm{c}}}^{\ast }})}^{\frac{2}{3}}{n}^{\ast -\frac{4}{3}},$$

where *ρ*_m_ denotes the number density of the melt, *n* denotes number of atoms in crystal nucleus, and ${n}^{\ast }={n}_{r={r}^{\ast }}=\frac{4}{3}\pi {r}^{\mathrm{\ast 3}}{\rho }_{{\rm{c}}}^{\ast }$. Additionally, ${\rho }_{{\rm{c}}}^{\ast }$ denotes atomic number density of the critical crystal nucleus. Furthermore, *f* ^+^(n*) denotes the attachment rate of atoms to the critical crystal nucleus, and *Z* denotes the Zeldovich factor^30^. The attachment rate *f* ^+^(n*) can be expressed as^31^ follows:

20 $${f}^{+}({n}^{\ast })=\frac{1}{2}\frac{\langle {\rm{\Delta }}{n}^{\mathrm{\ast 2}}(t)\rangle }{t},$$

where 〈Δn*^2^(*t*)〉 denotes the mean square deviation (MSD) of the atoms attached to the critical crystal nucleus during a time interval *t*. In the study, the nucleation rates *J* and incubation time *t* = 1/*J* are estimated using Eq. (8) with Eq. (17) using the atomistically determined ΔG* and *f* ^+^(n*). The TTT diagram is then depicted using the obtained incubation time *t* at different temperatures below the melting temperature *T*_m_.

## Electronic supplementary material

Supplementary Information
