# Supplementary material for: Predictive modeling of Time-Temperature-Transformation diagram of metallic glasses based on atomistically-informed classical nucleation theory
Source: Sci Rep. 2017 Aug 3;7:7194. doi: 10.1038/s41598-017-06482-8 (PMC5543070; doi:10.1038/s41598-017-06482-8)
Supplement: Supplementary file 1 — Supplementary Information [file 41598_2017_6482_MOESM1_ESM.pdf]

Supplementary Information

Predictive modeling of Time-Temperature-Transformation diagram of metallic glasses based on atomistically-informed classical nucleation theory

Yuji Sato[1], Chiaki Nakai[1], Masato Wakeda[1,*], Shigenobu Ogata[1,2,**]

[1]Department of Mechanical Science and Bioengineering, Osaka University, 1-3 Machikaneyama, Toyonaka, Osaka, 560-8531, Japan, [2]Center for Elements Strategy Initiative for Structural Materials (ESISM), Kyoto University, Sakyo, Kyoto, 606-8501, Japan

[*]wakeda@me.es.osaka-u.ac.jp
[**]ogata@me.es.osaka-u.ac.jp

Derivation of time dependent 1D temperature distribution $T(x,t)$

Time dependent temperature distribution $T(x,t)$ in an infinite plate is derived by solving the heat conduction equation:

$$\frac{\partial T}{\partial t} = \alpha \frac{\partial^2 T}{\partial x^2}, \tag{S1}$$

under the following initial and boundary conditions.

$$T(x,0) = T_\mathrm{i}, \tag{S2}$$

$$T(0,t) = T(L,t) = T_\mathrm{s}. \tag{S3}$$

Define a dimensionless temperature

$$\tilde{T}(x,t) = \frac{T(x,t) - T_\mathrm{s}}{T_\mathrm{i} - T_\mathrm{s}}. \tag{S4}$$

Eqs. (S1) $\sim$ (S3) are rewritten as:

$$\frac{\partial \tilde{T}}{\partial t} = \alpha \frac{\partial^2 \tilde{T}}{\partial x^2}, \tag{S5}$$

$$\tilde{T}(x,0) = 1, \tag{S6}$$

$$\tilde{T}(0,t) = \tilde{T}(L,t) = 0. \tag{S7}$$

Using the variable separation:

$$\tilde{T}(x,t) = X(x)U(t), \tag{S8}$$

Eq. (S5) is rewritten as:

$$X \frac{\mathrm{d}U}{\mathrm{d}t} = \alpha U \frac{\mathrm{d}^2 X}{\mathrm{d}x^2} \tag{S9}$$

$$\Leftrightarrow \quad \frac{1}{\alpha}\frac{1}{U}\frac{\mathrm{d}U}{\mathrm{d}t} = \frac{1}{X}\frac{\mathrm{d}^2 X}{\mathrm{d}x^2}. \tag{S10}$$

When Eq. (S10) holds, both sides of Eq. (S10) should be constant. In addition, if these are positive, U diverges to ∞ at $t \to \infty$. Hence, both sides of Eq. (S10) should be negative constant, and thus these can be expressed by a constant $\beta (> 0)$:

$$\frac{1}{\alpha}\frac{1}{U}\frac{\mathrm{d}U}{\mathrm{d}t} = \frac{1}{X}\frac{\mathrm{d}^2X}{\mathrm{d}x^2} = -\beta^2. \tag{S11}$$

Therefore, the following equations hold:

$$\begin{cases} \dfrac{\mathrm{d}U}{\mathrm{d}t} = -\beta^2\alpha U \\ \dfrac{\mathrm{d}^2X}{\mathrm{d}x^2} = -\beta^2 X \end{cases}. \tag{S12}$$

The general solution of Eq. (S12) is expressed as follows:

$$\begin{cases} U(t) = D_1\exp(-\beta^2\alpha t) \\ X(x) = D_2\cos(\beta x) + D_3\sin(\beta x) \end{cases}. \tag{S13}$$

Hence, $\tilde{T}$ is derived by plugging in Eq. (S13) for Eq. (S8):

$$\tilde{T} = \exp(-\beta^2\alpha t)(\tilde{D}_2\cos(\beta x) + \tilde{D}_3\sin(\beta x)), \tag{S14}$$

where $\tilde{D}_2 = D_1D_2, \tilde{D}_3 = D_1D_3$. Here, under the boundary condition (S7), the following equation holds:

$$\begin{cases} 0 = \tilde{D}_2\exp(-\beta^2\alpha t) \\ 0 = \tilde{D}_3\exp(-\beta^2\alpha t)\sin(\beta L) \end{cases}. \tag{S15}$$

By Eqs. (S14) and (S15),

$$\tilde{D}_2 = 0, \quad \sin(\beta L) = 0. \tag{S16}$$

Therefore, β is expressed by using a natural number p as follows:

$$\beta = \frac{p\pi}{L}, \tag{S17}$$

and by Eq. (S14), for all p, following equation satisfies Eqs. (S5) and (S7):

$$\tilde{T} = \tilde{D}_3\exp\left\{-\alpha\left(\frac{p\pi}{L}\right)^2 t\right\}\sin\left(\frac{p\pi}{L}x\right). \tag{S18}$$

Since Eqs. (S5) and (S7) are linear, the linear combination of Eq. (S18),

$$\tilde{T} = \sum_{p=1}^{\infty} D_3^{(p)}\exp\left\{-\alpha\left(\frac{p\pi}{L}\right)^2 t\right\}\sin\left(\frac{p\pi}{L}x\right), \tag{S19}$$

is also satisfies them. Eventually, $D_3^{(p)}$ is determined to satisfy Eq. (S6). By Eq. (S14), following equation holds:

$$1 = \sum_{p=1}^{\infty} D_3^{(p)}\sin\left(\frac{p\pi}{L}x\right), \tag{S20}$$

Then multiplying both sides of Eq. (S20) by $\sin(\frac{m\pi}{L}x)$ and integrating them for the interval 0 to L yield,

$$\begin{aligned}
(\text{left side of Eq. (S20)}) &= \int_0^L \sin\left(\frac{m\pi}{L}x\right) \mathrm{d}x \\
&= \left[-\frac{L}{m\pi}\cos\left(\frac{m\pi}{L}x\right)\right]_0^L \\
&= \frac{L}{m\pi}\left\{(-1)^{m+1}+1\right\}, \qquad \text{(S21)}
\end{aligned}$$

$$\begin{aligned}
(\text{right side of Eq. (S20)}) &= \sum_{p=1}^{\infty} D_3^{(p)} \int_0^L \sin\left(\frac{p\pi}{L}x\right)\sin\left(\frac{m\pi}{L}x\right) \mathrm{d}x \\
&= \sum_{p=1}^{\infty} D_3^{(p)} \frac{L}{2}\delta_{pm} \\
&= \frac{L}{2} D_3^{(m)}, \qquad \text{(S22)}
\end{aligned}$$

where δ denotes Kronecker delta. Hence, by Eqs. (S21) and (S22), $D_3^{(m)}$ is determined as follows:

$$D_3^{(m)} = \begin{cases} 0 & (m \text{ is even.}) \\ \dfrac{4}{m\pi} & (m \text{ is odd.}) \end{cases} . \qquad \text{(S23)}$$

Plugging in Eq. (S23) for Eq. (S19) yields:

$$\begin{aligned}
\tilde{T}(x,t) &= \sum_{p=1}^{\infty} \frac{4}{p\pi} \exp\left\{-\alpha\left(\frac{p\pi}{L}\right)^2 t\right\} \sin\left(\frac{p\pi}{L}x\right) \\
&= \sum_{m=0}^{\infty} \frac{4}{(2m+1)\pi} \exp\left[-\alpha\left\{\frac{(2m+1)\pi}{L}\right\}^2 t\right] \sin\left\{\frac{(2m+1)\pi}{L}x\right\}, \qquad \text{(S24)}
\end{aligned}$$

and then, $T(x,t)$ can be derived by plugging in Eq. (S24) for Eq. (S4).

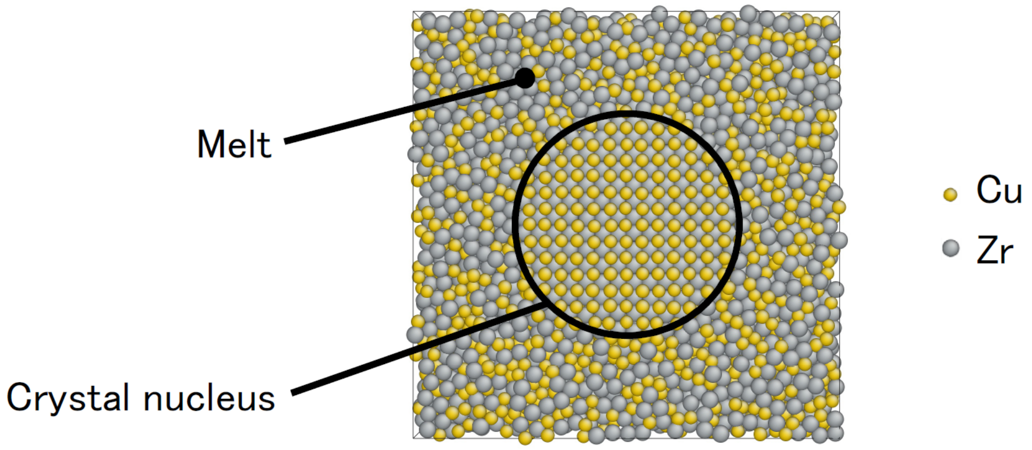

Supplementary Figure S1 : An example of a simulation model used for critical nucleation radius analysis ($Cu_{50}Zr_{50}$).

Supplementary Table S1 : Models for crystal growth or shrink simulations for $Cu_{50}Zr_{50}$. *r* and *n* denote the radius and the number of atoms, respectively, in the embedded crystal nucleus, *l* denotes the model cell dimension, and *N* denotes the total number of atoms in the model.

r (nm)	*n*	*l* (nm)	*N*
1.10	331	4.53	5,299
1.35	600	5.18	7,912
1.50	869	5.85	11,438
1.65	1,101	6.17	13,396
1.80	1,449	6.49	15,610
2.00	1,959	7.82	27,226
2.20	2,667	8.49	34,788
2.50	3,831	9.14	43,422
3.00	6,669	10.45	65,065
3.50	10,642	11.78	93,062

Supplementary Table S2 : Models for crystal growth or shrink simulations for $Cu_{20}Zr_{80}$. *r* and *n* denote the radius and the number of atoms, respectively, in the embedded crystal nucleus, *l* denotes the model cell dimension, and *N* denotes the total number of atoms in the model.

r (nm)	*n*	*l* (nm)	*N*
1.10	266	4.84	5,312
1.35	469	5.52	7,916
1.50	685	6.23	11,394
1.65	887	6.56	13,362
1.80	1,183	6.91	15,641
2.00	1,630	8.33	27,227
2.20	2,086	9.01	34,589
2.50	3,174	9.69	43,346
3.00	5,479	11.09	64,844
3.50	8,683	12.44	92,243
4.00	12,579	15.25	168,732

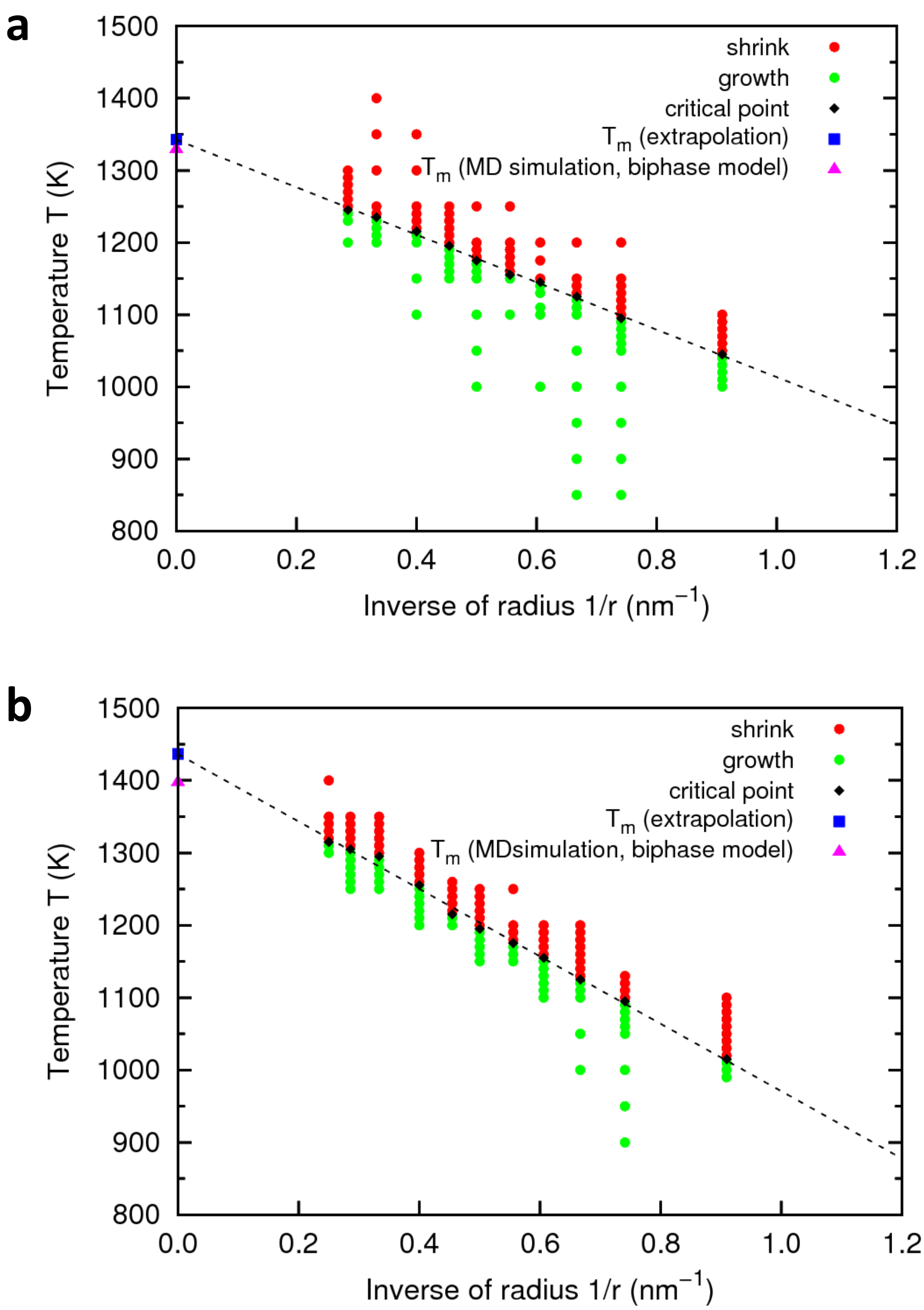

Supplementary Figure S2 : The relationship between temperature and inverse of radius ((a) $Cu_{50}Zr_{50}$, (b) $Cu_{20}Zr_{80}$). Estimated melting temperatures by extrapolation and direct MD simulation using biphase model (see Figure S3) are also shown by square and triangle plots, respectively.

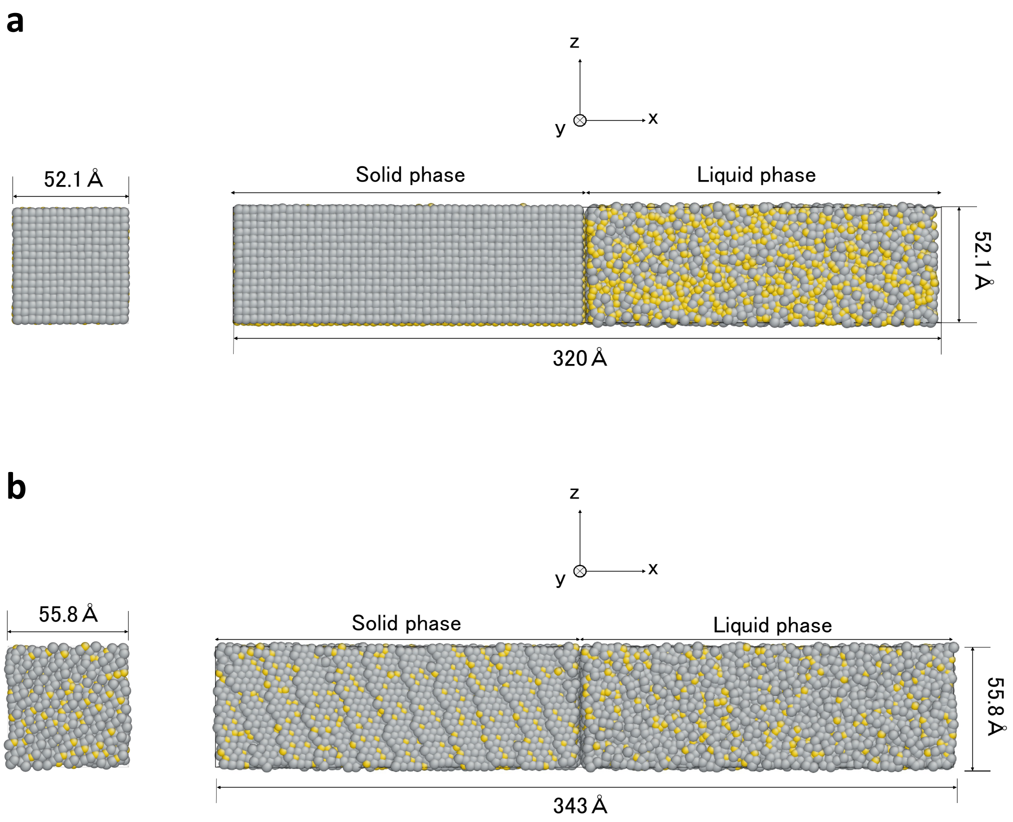

Supplementary Figure S3 : The melt-crystal biphase models ((a) $Cu_{50}Zr_{50}$, $N = 49,152$, (b) $Cu_{20}Zr_{80}$, $N = 51,200$).

Supplementary Table S3 : Obtained parameters for the fitting function in Eq. (4).

alloy	phase	a ($Jm^{-3}K^{-2}$)	b ($Jm^{-3}K^{-1}$)	c (Jm^{-3})
$Cu_{50}Zr_{50}$	crystal	1.71×10^{2}	3.38×10^{6}	-4.75×10^{10}
〃	liquid	1.34×10^{3}	2.58×10^{6}	-4.49×10^{10}
$Cu_{20}Zr_{80}$	crystal	5.14×10^{2}	2.78×10^{6}	-4.51×10^{10}
〃	liquid	1.07×10^{3}	2.20×10^{6}	-4.43×10^{10}

Supplementary Table S4 : Parameters obtained in Eq. (6).

alloy	A	B
$Cu_{50}Zr_{50}$	3.0×10^{11}	2.67×10^{4}
$Cu_{20}Zr_{80}$	3.8×10^{3}	3.57×10^{3}

a

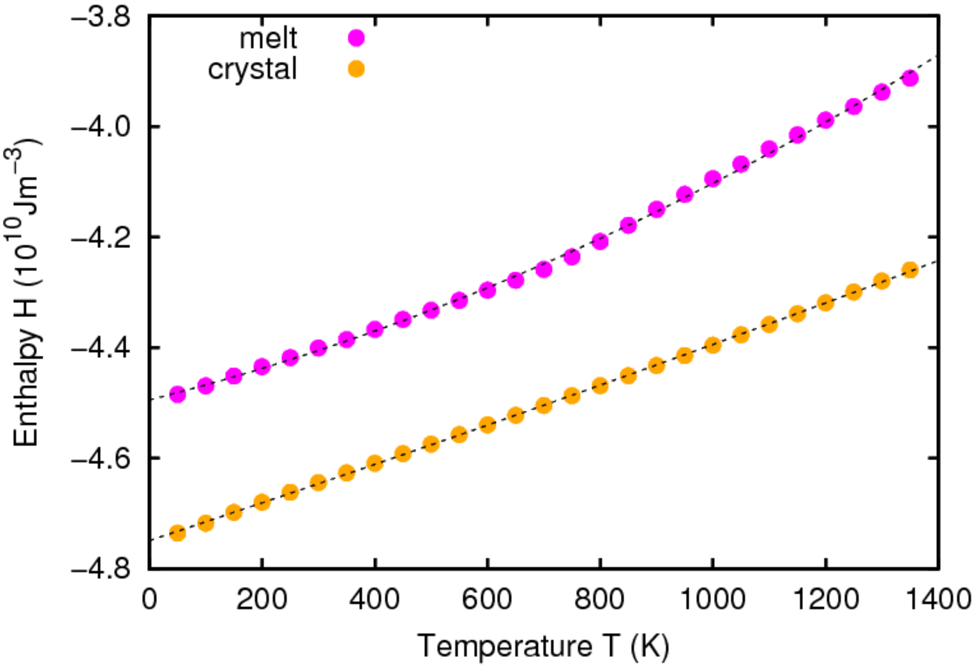

b

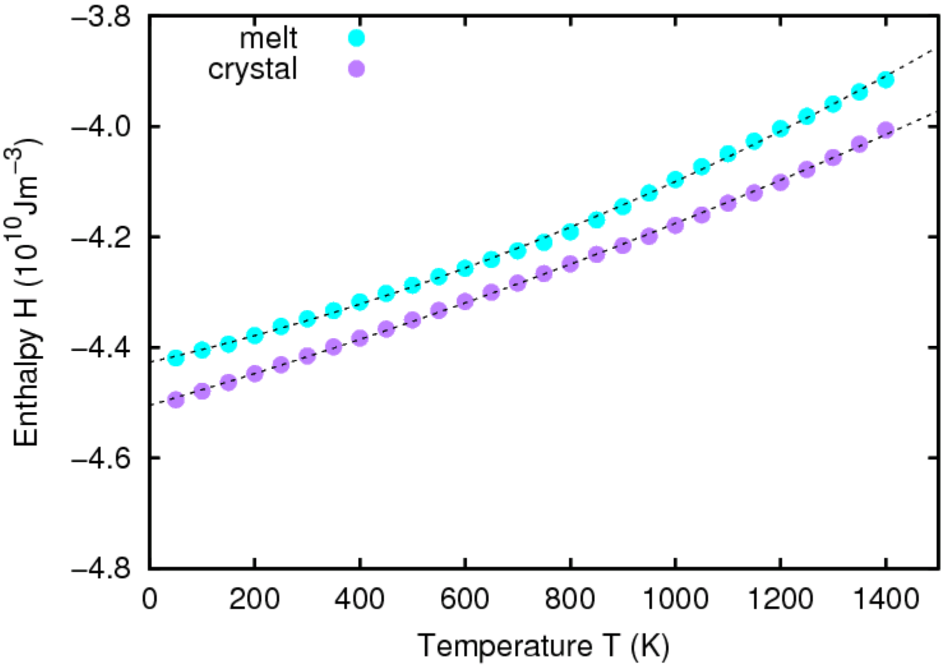

Supplementary Figure S4 : Temperature dependent enthalpies of the melt and crystal ((a) $Cu_{50}Zr_{50}$, (b) $Cu_{20}Zr_{80}$). Eq. (4) is plotted as a broken line.

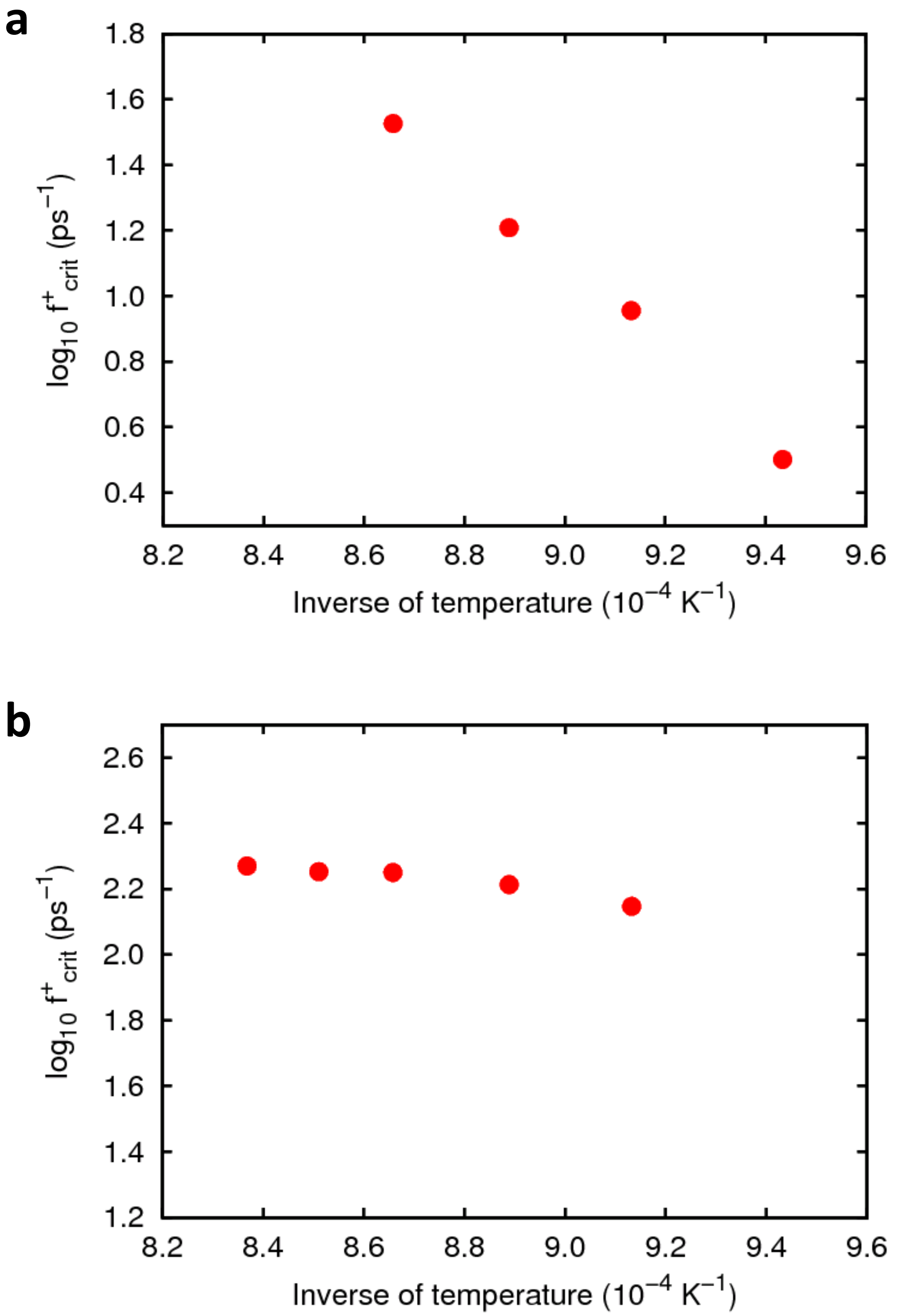

Supplementary Figure S5 : Arrhenius plot of attachment rate ((a) $Cu_{50}Zr_{50}$, (b) $Cu_{20}Zr_{80}$).

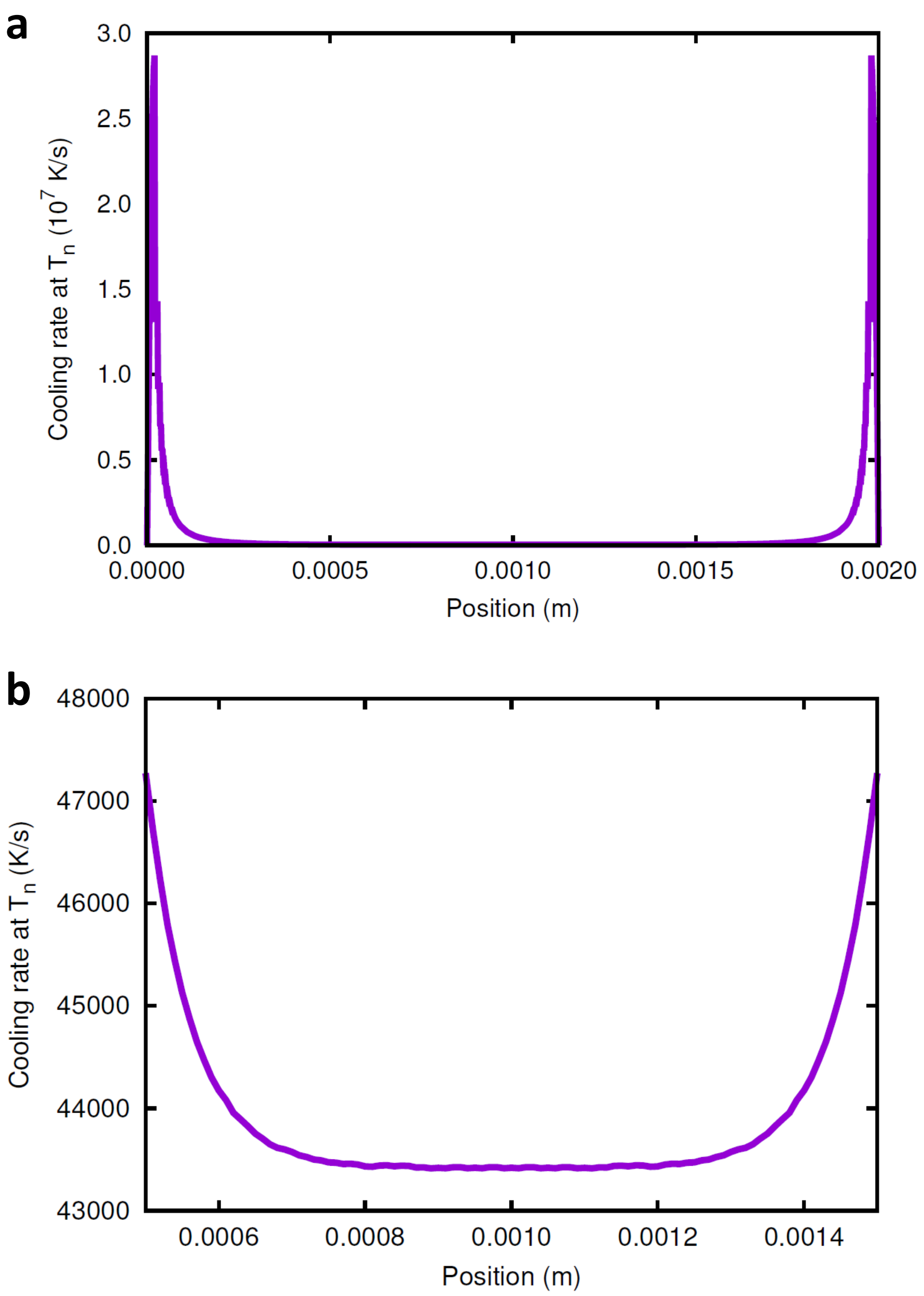

Supplementary Figure S6 : (a) The spatial distribution of cooling rate when $T(x,t_n) = T_n$. (b) Magnified view of (a). The minimum cooling rate was estimated as 4.34×10^4 K/s at $t_n = 1.24 \times 10^{-2}$ s and $x = L/2 = 0.0010$ m.
